# Efficacy of plasma activated saline in a co-culture infection control model

**DOI:** 10.1038/s41598-022-20165-z

**Published:** 2022-11-23

**Authors:** Evanthia Tsoukou, Paula Bourke, Daniela Boehm

**Affiliations:** 1 Environmental Sustainability and Health Institute, Technological University Dublin, Dublin 7, Ireland; 2 School of Food Science and Environmental Health, Technological University Dublin, Dublin 7, Ireland; 3 Plasma Research Group, School of Biosystems and Food Engineering, University College Dublin, Dublin 4, Ireland; 4 Conway Institute, University College Dublin, Dublin 4, Ireland

**Keywords:** Cell biology, Microbiology

## Abstract

Plasma activated liquids have demonstrated antimicrobial effects and receive increasing attention due to the potential to strengthen the armoury of novel approaches against antibiotic resistant bacteria. However, the antibacterial activity and cytotoxic effects of these solutions need to be understood and balanced before exposure to humans. In this study, the antibacterial effects of plasma activated saline (PAS) were tested against Gram negative and positive bacteria, and HaCaT keratinocytes were used for cytotoxicity studies. For the first time, a co-culture model between these bacteria and eukaryotic cells under the influence of PAS has been described. Exposure of saline to plasma resulted in high concentrations of nitrate, hydrogen peroxide and a reduction of pH. PAS caused high antibacterial effects in the co-culture model, accompanied by high cytotoxic effects to the monolayer of mammalian cells. We present evidence and provide a deeper understanding for the hypothesis that upon treatment with PAS, chemical species generated in the liquid mediate high antimicrobial effects in the co-culture setup as well as mitochondrial depolarization and glutathione depletion in HaCaT cells and cell lysis due to acidic pH. In conclusion, PAS retains strong antibacterial effects in a co-culture model, which may have unintended negative biological effects on mammalian cells.

## Introduction

The increasing morbidity of diseases such as metabolic disorders and diabetes, healing of chronic wounds and skin repair represent a big challenge in healthcare. During the wound-healing phase, keratinocyte cells have both regulatory and secretory functions, thus are the main players for re-epithelization of human skin^1^. Unsuccessful wound healing permits development of infections around the wound surface, initiation of inflammation, pain, and an increase of pH at the wound surface. For this reason, topical antibiotics and antiseptic solutions which are indicated in both the prevention and the treatment of infection are being used in clinics. However, the rising occurrence of bacteria resistant to conventional treatments is a cause of major concern and driving factor for exploring novel antibacterial strategies. Factors involved in the increasing rates of bacterial resistance by usage of topical antimicrobials include usage of low concentrations, extended use in the physician practices, and prolonged period of therapy. In response to rising rates of resistance to conventional antimicrobial agents, new topical agents are advised not to be used for a long duration and should be replaced by a wound dressing once the bacterial burden is reduced^2^.

Despite the generation of several advanced wound healing agents to promote the healing of chronic wounds such as advanced dressings and polymers^3–5^, research on the applicability of other potentially useful topical agents which display bactericidal activity for the treatment of wound infections is needed to target bacterial infections and combat antimicrobial resistance. Plasma medicine has emerged as a new technology alternative to antibiotic treatments. Cold atmospheric plasma is the fourth state of matter, it is a totally or partially ionized gas and consists of photons, electromagnetic and magnetic fields, UV rays, electrons, ions and neutral radicals such as reactive oxygen and nitrogen species^6^. Cold plasma has been used clinically for applications such as sterilization or decontamination against bacteria, wound healing and cancer treatment^7,8^. Attempts to modify a liquid via plasma exposure and/or to generate plasma in liquids have been successful and plasma-activated liquids (PAL), also known as plasma treated, plasma functionalized or plasma conditioned liquids, have been introduced as an effective solution that can be applied in living tissues and cancer cells^9^ and may have several benefits over exposure to direct plasma. PAL can be stored at low temperatures over a period of months and still retain their antibacterial effects^10^, can be easily transported and can be delivered in a range of modalities to reach the internal cavities of the human body.

PAL offers a novel technology which could be used instead of or in conjunction with antibiotics, disinfectants, or antiseptics for biological tissues and subsequently for wound healing. PAL have shown inactivation effects on different strains of planktonic bacteria, biofilms, viruses, spores and have been proposed as an alternative method of disinfection for medical devices such as duodenoscope reprocessing^11–15^. PAL are usually generated by atmospheric cold plasma discharges directly within the liquids, discharges in the gas phase over the liquid surface and multiphase discharges^16^. Changes in the physicochemical properties of PAL depend on plasma treatment time, working gas, nature of solution and plasma device^17^. Exposure of liquids to cold plasma result in aqueous solutions which consist of relatively long-lived reactive oxygen and nitrogen species such as hydrogen peroxide, nitrites, nitrates^12^ and other short lived chemical species such as nitric oxide, hydroxyl radicals, superoxide, peroxynitrate, and peroxynitrite^18^. Synergistic effects of long lived reactive chemical species in combination with low pH are believed to be the main factors for antibacterial effects of PAL^19–21^. Short lived chemical species may play important roles as aqueous hydrogen peroxide and nitrite at acidic pH can lead to the formation of ONOOH^22^. The biological effects of PAL against bacteria have been extensively studied and data have shown that PAL can cause changes in the structure of the cells, such as shrinkage and development of holes on the surface of bacterial cells, increase of intracellular RONS and leakage of intracellular components after treatment with PAL^23–25^. Understanding the mechanisms of PAL effects on bacteria is necessary in order to evolve this new technology effectively into disinfection strategies.

PAL application could be modulated to target multiple effects: cleaning the infected area of a wound and inactivation of bacteria in it and stimulation of tissue regeneration such as fibroblast and skin cell growth. The application of plasma technology in wound healing has been studied for stimulation of epithelial and immune cells. Plasma increases translation of genes related to wound healing such as cytokines and growth factors and potentially promotes angiogenesis, cell adhesion or proliferation with subsequent promotion of the wound healing process^26^. Exposure to plasma can also result in high levels of DNA damage, cytotoxic effects leading to rapid necrotic cell death with H_2_O_2_ playing a key role in these effects^27^.

The primary aim of this study was to ascertain the relationship between antimicrobial efficacy and application safety of plasma activated saline (PAS), as a biomedically relevant solution for disinfection and thus its potential for use as an antiseptic or decontamination solution. Effects on a range of clinically relevant challenge bacteria were assessed. Mammalian cell damage was investigated by examination of the HaCaT keratinocyte cell line as an indication of the effects on the skin and potential risks to a patient. An in vitro co-culture model of the keratinocyte cell line and bacteria is presented. The microbial inactivation efficacy in the presence of a keratinocyte cell layer was assessed, and the cell death pathway and oxidative stress of HaCaT cells after exposure to PAS at different dilutions were investigated to identify if a balance between cytotoxic and microbial inactivation effects could be achieved in a co-culture model.

## Materials and methods

### Plasma system setup

Two electrical discharge configurations are achievable with this custom plasma system which is operated in atmospheric air and has been characterized in detail^28–30^. Characteristics of the electrical discharge and OES measurements have been reported in Lu et al., and Ng et al. A stainless-steel needle served as the high voltage electrode and it was fixed perpendicular to the liquid surface. The distance between the high voltage needle tip and the liquid surface was fixed to 5 mm for all experiments.

**Figure 1:**
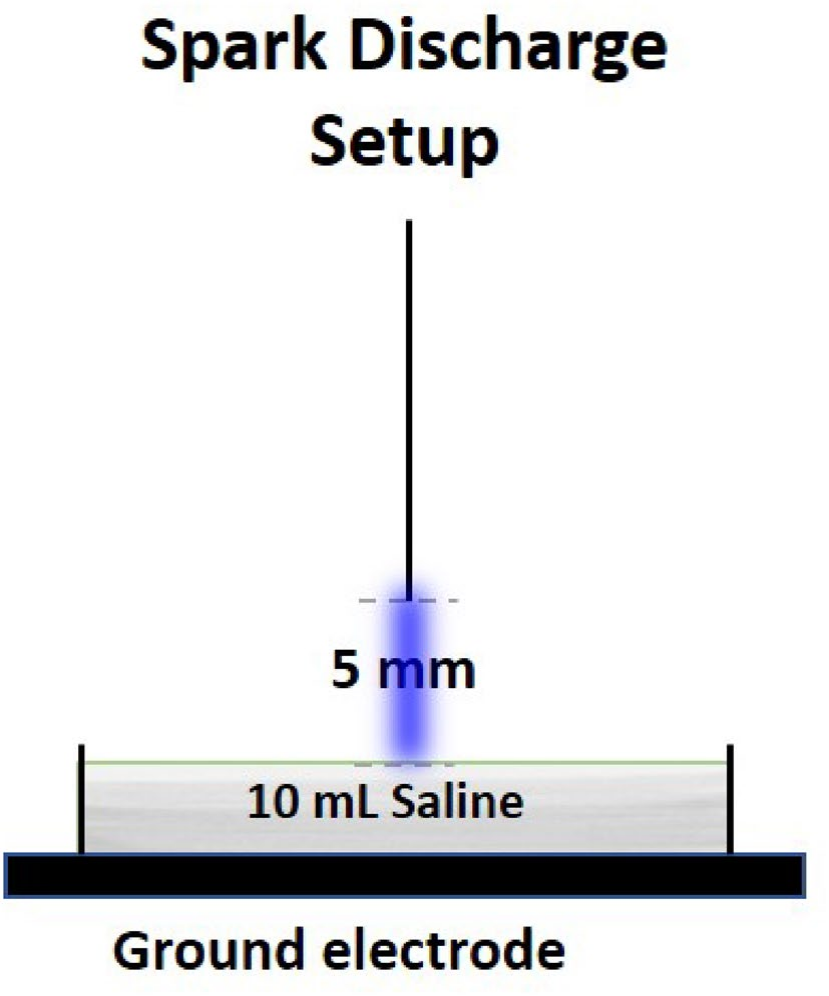
Spark discharge setup configuration.

For the spark setup (Fig. 1), the plastic petri dish was placed on a stainless-steel plate which was connected to the ground. Its maximum output voltage was 20 kV with a variable frequency of 20–65 kHz depending on the plasma load capacitance but in this study a fixed frequency of 25 kHz was used. For the generation of PAS, 10 mL volume sterile saline was added into a plastic petri dish (55 mm inner diameter), which corresponded to a liquid layer of about 4.2 mm depth. The liquids were exposed to plasma for up to 20 or 30 min, using atmospheric air. Samples were removed at regular intervals for analysis. The power supply used for driving plasma discharges was a HV half bridge resonant inverter circuit (PVM500, Information Unlimited).

### Investigation of the chemical composition of PAS

#### Quantification of hydrogen peroxide using titanium oxysulfate

Hydrogen peroxide concentrations were quantified as described previously^10^ employing the titanium oxysulfate (TiOSO_4_, Sigma-Aldrich, Arklow, Ireland) colorimetric method by incubating 10 µL TiOSO_4_ to 100 µL of PAS in the dark for ten minutes producing the yellow pertitanic acid. This absorbance was measured at 405 nm using a spectrophotometric microplate reader (VarioSkan Lux or MultiSkan GO, ThermoScientific, Waltham, MA, USA). Standard curves of known hydrogen peroxide were included on each plate and used to convert absorbance into concentrations.

#### Quantification of peroxides using potassium iodine

The concentrations of peroxide/oxidative species in PAS were determined by oxidation of potassium iodide to yellow iodine and spectrophotometric measurement. 50 µL of PAS or standard curve (H_2_O_2_) samples were added to 50 µL of phosphate buffer solution (10 mM) (buffered condition) or deionised water (non-buffered condition) and 100 µL 1 M potassium iodide (Sigma-Aldrich, Arklow, Ireland) in a 96 well microtiter plate at room temperature, incubated for twenty minutes and the absorbance was read at 390 nm^31^. Incubation with water in place of phosphate buffer allows for the detection of peroxides/oxidative species which are not stable at neutral pH^24^.

#### Determination of nitrite and nitrate

Concentration of nitrites was determined using Griess reagent (Sigma-Aldrich, Arklow, Ireland) as described previously^10^. A total of 50 µL of Griess reagent was added to 50 µl of PAS/standard curve sample. Absorbance was read at 548 nm, after 30 min of incubation, and compared to a sodium nitrite standard curve. A range of known concentrations of sodium nitrite was used to prepare the nitrite calibration curve and to convert absorbance into nitrite concentration.

Nitrate concentrations were determined photometrically by 2,6-dimethyl phenol (DMP) using the Spectroquant® nitrate assay kit (Merck Chemicals, Darmstadt, Germany) adapted to a 96-well plate format as described previously^10^. Sulfamic acid was used for pre-treatment of PAS for elimination of nitrite interference. 200 µL of reagent A, 25 µL of treated sample and 25 µL reagent B was added into a microtube and incubated for 20 min. After incubation period, 100 µL of the total mixture was added to a fresh 96 well plate microtiter plate and absorbance was read at 340 nm in duplicate.

#### pH measurements

The pH of PAS was measured by an Orion pH meter (model 420A, Thermo Electron Corporation, USA) after plasma treatment of the solutions. Acidic control solutions were prepared by dilutions of HCl (Sigma-Aldrich, Arklow, Ireland).

### Antimicrobial efficacy

#### Test organisms and growth conditions

*Escherichia coli* NCTC 12900, *S. aureus* ATCC 1803 and *S. aureus* ATCC 25923 were grown in tryptic soy broth (TSB, Scharlau Chemie, Spain) at 37 °C overnight. The first two strains are food pathogen bacteria, which were used for consistency and comparability with previous investigations of the same plasma device, whereas the last one is a biomedically relevant strain.

#### Chemical disinfectants

Common chemical disinfectants were tested as positive controls and to compare their antibacterial efficacy and cytotoxicity with PAS. Iodine 10% w/w Cutaneous Solution was purchased from a local pharmacy and was diluted in sterile saline according to manufacturer to make 1% povidone iodine solution. Hibiscrub 4% w/v chlorhexidine cutaneous solution was bought from a local pharmacy and diluted to 1% chlorhexidine in water. 0.045% NaClO was made by dilution in water of bleach consisting of 4.5% w/w sodium hypochlorite. 2% formaldehyde, 2% glutaraldehyde, 70% ethanol and 3% H_2_O_2_ were prepared through dilution in water of solutions purchased from Sigma (Sigma-Aldrich, Arklow, Ireland).

#### Preparation of bacterial cell suspensions

The cells were harvested from over-night cultures by centrifugation at 10,000 rpm for 5 min, washed three times in PBS and finally re-suspended in PBS. *E. coli* or *S. aureus* suspensions (30μL) were added to 970 μl of PBS, to form the bacterial working solutions. For the determination of each plasma activated solution’s antimicrobial effect, 10% bacterial suspension was added to 90% PAS/disinfectant and incubated at room temperature for 15, 30, 40 and 60 min (contact time).

#### Microbiological analysis

Microbiological analysis was performed as described previously^10^: after each contact time, a concentrated PBS (4.5xPBS concentration) solution was added to the bacterial solution to neutralize the pH. Samples were serially diluted in Maximum Recovery Diluent (MRD; Merck, Ireland) and 10 µL droplets were placed on TSA plates in triplicate. The plates were incubated aerobically at 37 °C for 24 h, after which colonies were counted to determine the number of viable cells. Results obtained are represented as surviving bacterial population in log_10_ colony forming unit (CFU/mL).

### Cell culture

#### Eukaryotic cell line

The cytotoxicity of PAS was examined using the immortal human keratinocyte (HaCaT) cell line obtained from Prof Fiona Lyng (Radiation and Environmental Science Centre, Technological University Dublin). HaCaT cells were cultured in Dulbecco’s modified Eagle’s medium/Ham's F-12 Nutrient Mixture (DMEM/F12, Sigma-Aldrich, Arklow, Ireland), supplemented with 2 mM l-glutamine and 10% (v/v) foetal bovine serum (FBS, Sigma-Aldrich, Arklow, Ireland). Cells were grown at 37 °C and 5% carbon dioxide (CO_2_) in a humidified incubator and sub-cultured using trypsin–EDTA (Sigma-Aldrich, Arklow, Ireland). Cell concentrations and viability were assessed using trypan blue counting.

HaCaT cells were seeded at a density of 5 × 10^6^ cells/mL into 96 well cell culture plates in 100 μL/well DMEM supplemented with foetal bovine serum (FBS; 10%) and l-glutamine, to reach a confluent layer after 24 h. The cell culture medium was removed and 100 μL of PAS were added to each well for 15–60 min contact time at room temperature. After each interval time, PAS was removed, the wells were washed with sterile phosphate buffer solution (PBS) and fresh medium was added to the wells and incubated in a humidified atmosphere of 5% CO_2_ at 37 °C. Three replicate wells per contact time were examined.

#### Viability assay

Cell viability was analysed using the resazurin dye (Sigma-Aldrich, Arklow, Ireland), a redox indicator that generates fluorescent signal by metabolic reduction and the reducing environment of living cells causes the indicator to change colour from the blue oxidized-form to the red reduced form. For resazurin solution 10 mg/mL resazurin sodium salt was dissolved in PBS and 8 μL/mL of this solution was diluted in DMEM/F12 without FBS. After culture supernatant or PAL were aspirated, wells were washed with warm PBS, 100 μL resazurin solution was added to each well and plates were incubated for 2 h at 37 °C. Absorbance was measured at 570 nm and 600 nm using a plate reader.

#### Flow cytometry analysis of apoptosis/necrosis

HaCaT cells were seeded at a density of 5 × 10^6^ cells/mL into 6 well cell culture plates in 2 mL media, under same conditions as described above. Annexin V-7AAD apoptosis detection kit was used as described by the manufacturer in dilution 1:10 for AnnV (Thermofischer, Massachusetts, United States). After PBS wash, the cells were stained and then analysed by flow cytometry (CytoFLEX, Beckmann Coulter, Brea, CA, USA).

#### Detection of intracellular reactive oxygen species

For intracellular ROS measurements, the cells were plated at a density of 5 × 10^6^ cells/mL and after 18 h of adherence, HaCaT cells were loaded with 100 μL 2′,7′-dichlorodihydrofluorescein diacetate (H_2_DCFDA, 50 μM) for 45 min at 37 °C and 5% CO_2_. Subsequent treatment with different percentages of PAS followed and intracellular ROS levels were then detected using a plate reader (excitation, 485 nm; and emission, 535 nm; VarioSkan Lux, ThermoScientific, Waltham, MA, USA). Triplicates were run for each condition.

#### Mitochondrial membrane potential analysis

The mitochondrial membrane potential was analysed by staining with JC-1. After 18 h adherence of cells seeded at 5 × 10^6^ cells/mL, media was removed and cells were washed with warm PBS and PAS treatment followed. 100 μL of 2 μM of JC-1 dye were added and plates were incubated at 37 °C, 5% CO_2_ for 30 min. After incubation, the dye was removed, wells were washed and 100 μL of PBS were added to each well. Mitochondrial membrane potential was detected using a plate reader (Ex 535 nm, Em 595 for the aggregates and Ex 485 nm, Em 535 nm for the monomers; VarioSkan Lux, ThermoScientific, Waltham, MA, USA). The ratio was determined between the aggregates’ fluorescence and the monomers’ fluorescence. Analyses were performed in triplicate for each condition.

#### Glutathione measurements

Cellular concentrations of total and oxidised glutathione were analysed using a glutathione assay kit (Sigma-Aldrich, Arklow, Ireland). Cells were seeded and treated as mentioned before. For total glutathione measurements, samples were washed, trypsinised and collected in tubes, deproteinized with 5% sulfosalicylic acid (SSA) (Sigma) and diluted with assay buffer to 1% SSA according to manufacturer’s instruction (Thermofischer, Massachusetts, United States). To measure GSSH, samples were initially treated with 2-vinylpyridine solution (Sigma) following the same procedure. For measurements of neutralised solutions, PAS was treated with NaOH before incubation with HaCaT cells. Absorbance was measured utilizing a microplate reader at 405 nm (MultiSkan GO, ThermoScientific, Waltham, MA, USA).

#### Lipid peroxidation

Lipid peroxidation was assessed based on the detection of malonedialdehyde (MDA) using a lipid peroxidation (MDA) detection kit (Sigma-Aldrich, Arklow, Ireland). Cells were seeded, treated as described above and collected by trypsinisation. Cells were then centrifuged at 100 g for 5 min and homogenized by sonication (Sonics, vibracell, Connecticut, United States). 100µL homogenate were added into a tube with 200 µL ice cold 10% trichloroacetic acid and incubated for 15 min on ice and then samples were centrifuged at 2200 g for 15 min at 4 °C. Then, 200 µL supernatant or standards were added to 200 µL 0.67% (w/v) thiobarbituric acid (Sigma-Aldrich, Arklow, Ireland) and incubated in a boiling water bath for 10 min. The samples were then cooled for 10 min and 150 µL were transferred in 96 well plate and absorbance was read at 532 nm. Standards were prepared by different dilutions of 1,1,3,3-tetramethoxypropane (Sigma-Aldrich, Arklow, Ireland) from 0.625 to 50 µL.

### Co-culture model setup

#### Microbial inactivation in co-culture model

A confluent layer of HaCaT cells grown on 96 well plates was used. The cell culture medium was removed, and wells were washed with sterile PBS. Then, 10 μL of bacteria suspension was added to each well, followed by 90 μL of the test solution to each well. After each contact time (15, 30, 40 or 60 min), 30 μL 4.5 × sterile PBS was added to the bacterial solution and serial dilutions were then prepared in MRD; 10 μl of the liquid suspension of each dilution was plated in triplicate on TSA plates. The CFU were counted after 24–48 h of incubation at 37 °C.

#### Antibiotic Invasion assay

After microbial inactivation, the number of internalised bacteria was measured using the antibiotic invasion assay. The PAS supernatant (containing bacteria) was discarded, the wells were washed with sterile PBS and replaced with 200 μL DMEM without serum supplemented with 100 μg/mL gentamicin for *E. coli* or 10 μg/mL lysostaphin for *S. aureus*^32,33^. Plates were incubated at 37 °C in 5% CO_2_ for 1 h to inactivate all extracellular bacteria. After incubation, wells were washed with sterile PBS and 200 μL of 0.1% TritonX100. The cells were gently homogenized by repeat pipetting and incubated for 10 min at room temperature. Controls of wells containing only PAL and bacteria were similarly tested. After incubation, the liquid suspension was plated onto TSA as described above.

### Statistical analysis

Results are presented as the means of three independent experiments with standard deviations, prepared using GraphPad Prism version 5 (GraphPad Software Inc., San Diego, USA, https://www.graphpad.com). Comparisons between different groups were performed in GraphPad Prism by two-way ANOVA with Bonferroni post-test and levels of significance are displayed as * p < 0.05, ** p < 0.01, *** p < 0.001.

## Results

### Hydrogen peroxide and nitrates are the main species detected in PAS

**Figure 2:**
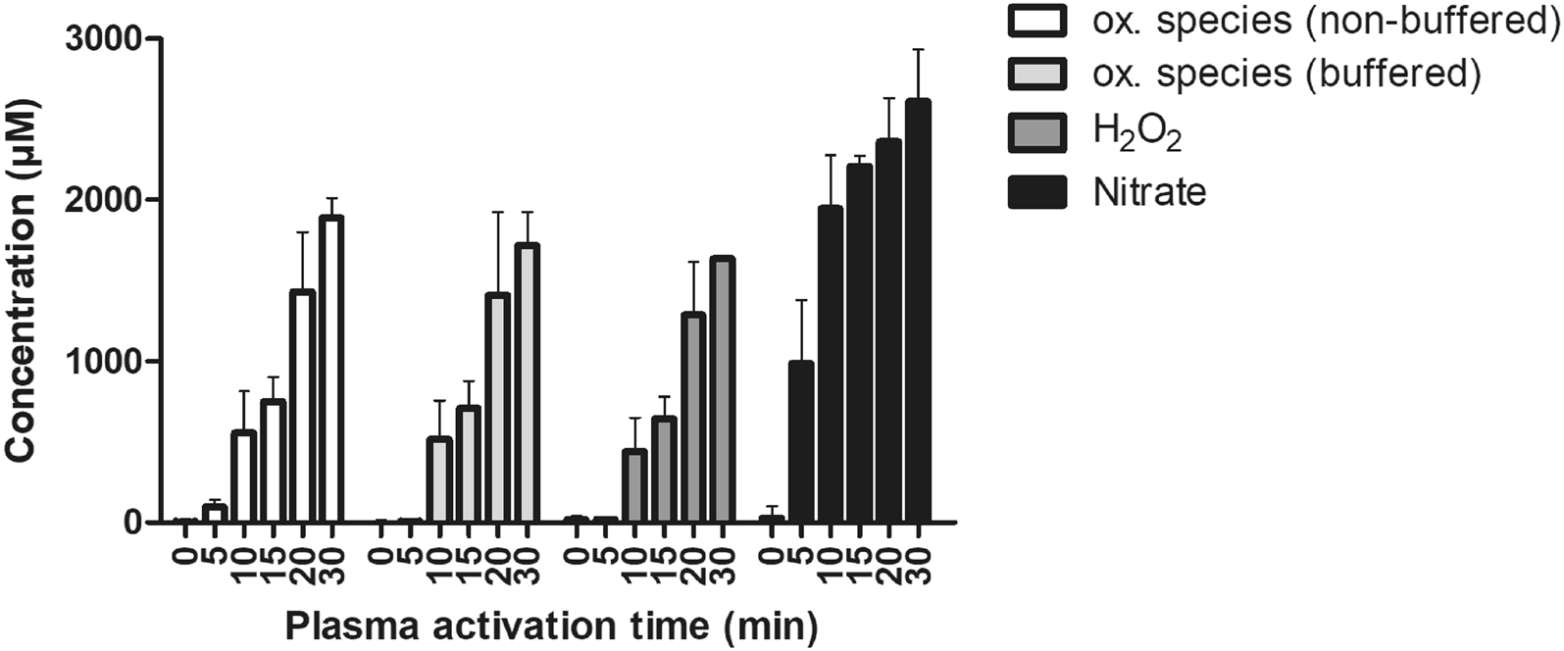
Chemical composition of PAS after exposure to spark plasma discharge for activation times of 0–30 min. Oxidative species refer to species able to oxidize potassium iodide (KI) at acidic pH (non-buffered) or neutral pH (buffered). The results are the mean ± S.D. of three determinations.

Plasma treatment in liquids leads to the generation of chemical species such as reactive oxygen and nitrogen species, which mediate subsequent plasma-induced effects on prokaryotic and eukaryotic cells. The concentration of hydrogen peroxide increased within saline as a function of plasma treatment time, where 17 μM was recorded after 5 min plasma treatment, reaching 643 μM after 15 min, 1282 μM after 20 min and 1632 μM after 30 min treatment time (Fig. 2). Similar trendlines to hydrogen peroxides were observed for total oxidative species measured under non-buffered or buffered conditions. Concentrations of oxidative species, stable at acidic pH (non-buffered conditions) or neutral pH (buffered conditions), respectively, were similar to hydrogen peroxide, indicating that almost all oxidative species generated and retained in PAS were hydrogen peroxide, which is in accordance with a previous study on PAW using the same plasma system^28^. Nitrate concentrations increased up to 900 μM after 5 min exposure to plasma and doubled after 10 min, with 2610 μM reached after 30 min plasma treatment time. No nitrites were detected in agreement with previous studies using the same plasma system.

### Antibacterial effects of PAS in a co-culture model

**Figure 3:**
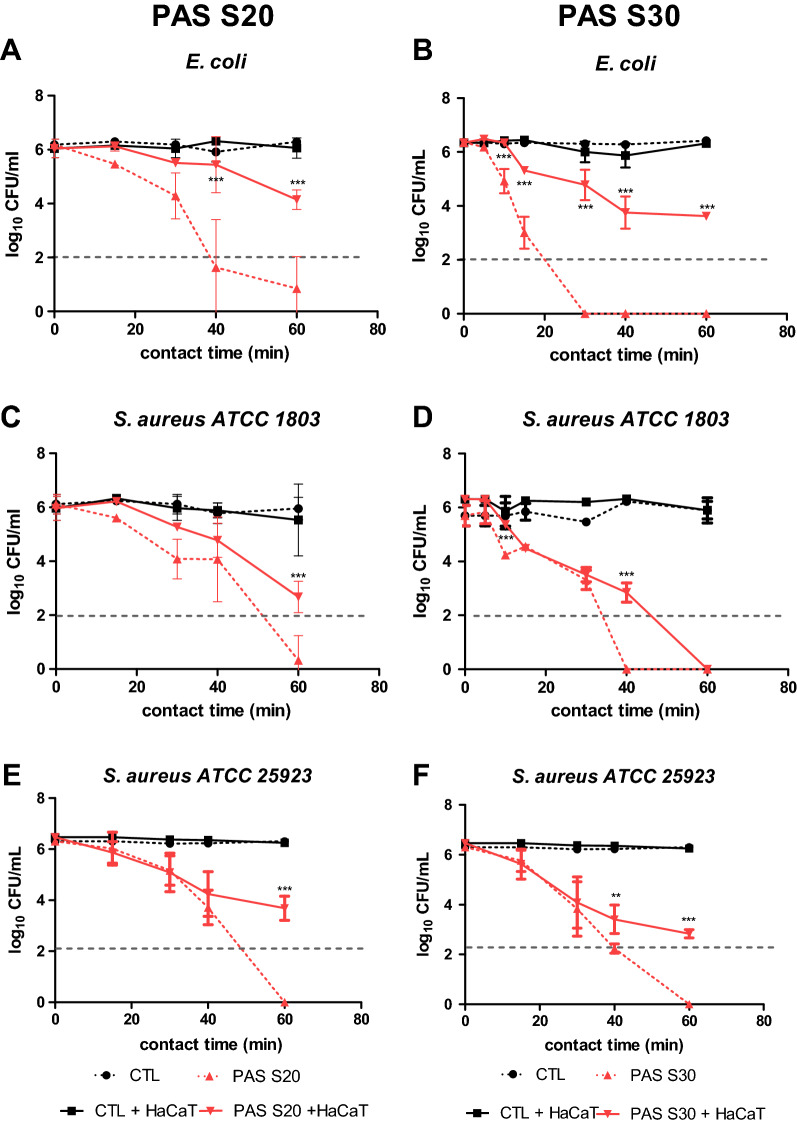
Bacterial inactivation in a co-culture model of HaCaT cells and *E. coli* or *S. aureus* under incubation with PAS 20 or PAS 30 as a function of contact time. (**A**) *E. coli*—PAS 20, (**B**) *E. coli*—PAS 30, (**C**) *S. aureus* ATCC 1803—PAS 20, (**D**) *S. aureus* ATCC 1803—PAS 30, (**E**) *S. aureus* ATCC 25923—PAS 20, (**F**) *S. aureus* ATCC 25923—PAS 30. The results are the mean ± S.D. of three determinations, limit of detection 2 log CFU/mL. Statistical significance is shown for comparison of bacteria + HaCaT cells (co-culture) incubated with PAS to only bacteria (no co-culture) incubated with PAS, with significance levels indicated as *** p < 0.001, ** p < 0.01, * p < 0.05.

The effect of PAS on both Gram-positive and Gram-negative bacteria, *Escherichia coli* and *Staphylococcus aureus*, was investigated. Initially PAS 10 and PAS 15 were tested for their antibacterial effects (data not shown) but due to limited antibacterial efficacy in the co-culture model, the plasma treatment time of saline was increased to 20 and 30 min (PAS 20 and PAS 30). Thereafter, the effect of contact time between bacteria and PAS was investigated. With regards to *E. coli*, PAS 20 was found to gradually reduce bacterial load as contact time increased, and a 6 log cycle reduction was achieved after 60 min contact time (Fig. 3A). In the co-culture model, the antibacterial efficacy was reduced, and PAS 20 was not able to reduce *E. coli* to the same extent, and only 2 log cycle reduction occurred at the longest contact time of 60 min (Fig. 3A). *S. aureus* ATCC 1803 was reduced by 2 log after 30 min contact time, and 6 log after 60 min contact time (Fig. 3C). PAS 20 retained better efficacy against the Gram positive challenge in the co-culture model where, in the presence of the HaCaT monolayer, *S. aureus* ATCC 1803 was reduced by 3 log within a 60 min contact time.

The microbial inactivation of PAS 30 was also investigated to assess if the contact time could be reduced. *E. coli* was inactivated by 6 log after 30 min contact time with PAS 30, which was 10 min earlier than PAS 20, but when PAS 30 was challenged with the co-culture, the microbial load was not further reduced, even with extended contact time (Fig. 3B). PAS 30 reduced *S. aureus* ATCC 1803 by 6 log cycles after 40 min contact time (20 min earlier than PAS 20), and this effect persisted in co-culture, where PAS 30 reduced the microbial load by 6 log at 60 min contact time (Fig. 3D). PAS 20 inactivation efficacy against *S. aureus* ATCC 25923 was similar to that observed for *S. aureus* ATCC 1803. However, PAS 30 led to 6 log reduction only after 60 min contact time and 3 log reduction in the co-culture set up (Fig. 3E, F).

### Antimicrobial efficacy in a co-culture model dependent on PAS dilution

**Figure 4:**
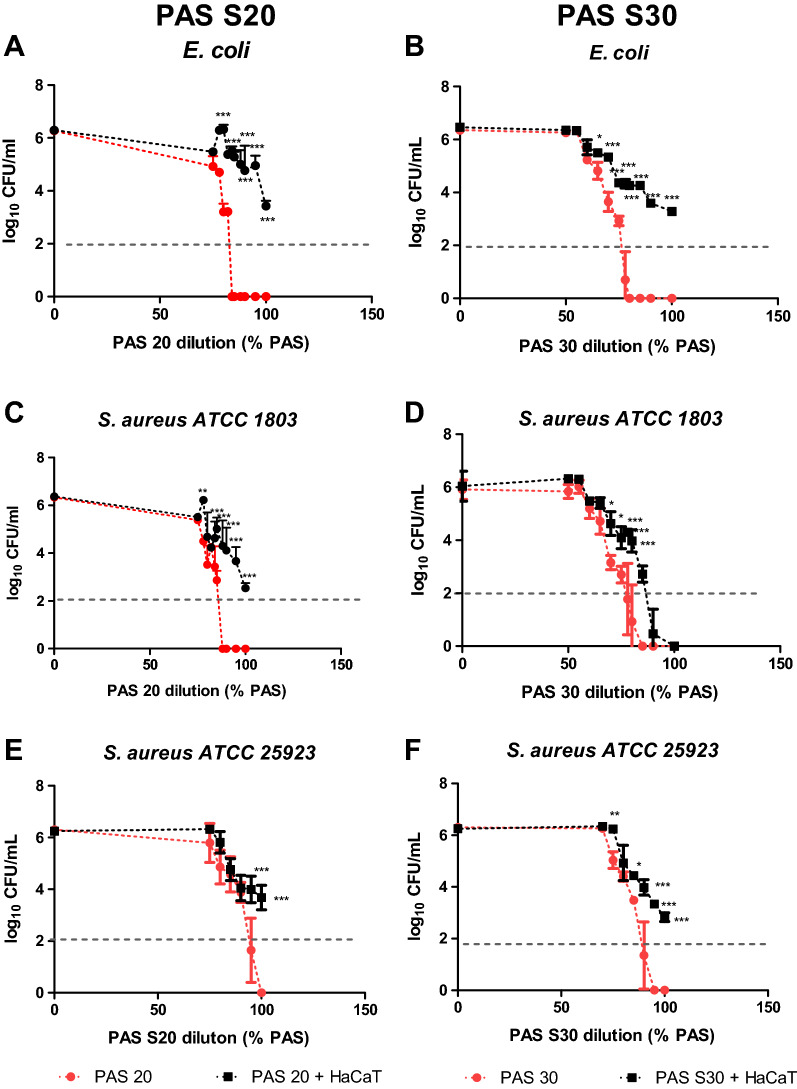
The antimicrobial efficacy of PAS 20 and PAS 30 within the co-culture model between HaCaT cells and *E. coli* or *S. aureus* and incubation with diluted PAS 20 or PAS 30 after 60 min contact time. (**A**) *E. coli*—PAS 20, (**B**) *E. coli*—PAS 30, (**C**) *S. aureus* ATCC 1803—PAS 20, (**D**) *S. aureus* ATCC 1803—PAS 30, (**E**) *S. aureus* ATCC 25923—PAS 20, (**F**) *S. aureus* ATCC 25923—PAS 30. The results are the mean ± S.D. of three determinations, limit of detection 2 log CFU/mL. Statistical significance is shown for comparison of bacteria + HaCaT cells (co-culture) incubated with PAS to only bacteria (no co-culture) incubated with PAS, with significance levels indicated as *** p < 0.001, ** p < 0.01, * p < 0.05.

A useful balance between minimal cytotoxic and efficient antimicrobial effects could make these solutions ideal candidates as disinfectants or antiseptics. To investigate if this scenario was feasible, dilutions ranging from 70 to 100% PAS were made in sterile PBS and then tested against the same bacteria strains for 60 min contact time as this was the most effective process for microbial inactivation. Figure 4 shows that 75% PAS 20 can cause 1.5 log reduction for *E. coli* suspensions, 80–82% PAS 20 yields a 3 log reduction and 84–100% PAS 20 a 6 log reduction (Fig. 4A). In contrast, when the bacteria were in the presence of a cell layer, a 6 log cycle inactivation was only achieved when 100% PAS was applied. *S. aureus* ATCC 1803 was reduced by approximately 3 log cycles when incubated with 85% PAS, whereas 88–100% PAS 20 solutions achieved 6 log reductions (Fig. 4C). In the co-culture model, *S. aureus* ATCC 1803 was reduced by 3.5 log by 100% PAS 20, whereas 95% PAS 20 caused 2.5 log reduction. *S. aureus* ATCC 25923 seemed to be slightly more resistant than *S. aureus* ATCC 1803 (Fig. 4E).

The same method was followed for PAS 30. Solutions of PAS 30 at higher than 78% and 80%, led to 6 log reduction for *E. coli* and *S. aureus*, respectively showing that PAS 30 has stronger antibacterial effects (Fig. 4B, D). The highest inactivation in the co-culture model was achieved with PAS 30 concentrations of 90% and 100%. A 2.5 log reduction was obtained for *E. coli* after 60 min*. E. coli* was further incubated up to 24 h in the co-culture setup to establish if inactivation was possible after longer contact time. A further decrease was noted after 4 h, with complete inactivation after 6 h of contact time (results not shown). When in co-culture, *S. aureus* was inactivated by 100% PAS 30, following the same trend as the inactivation trial without the monolayer. *S. aureus* ATCC 25923 was reduced by 6 log when incubated with 95% PAS 30 and 100% PAS 30, but the inactivation efficiency was reduced in the co-culture, and did not exceed 3 log cycles. The reduction factor decreased as PAS 30 dilution increased (Fig. 4F).

### Internalisation of bacteria to host cells

To determine whether the reduction of bacterial counts was indeed the result of inactivation by PAS, we next investigated if bacteria were internalized into host cells using the gentamicin/lysostaphin invasion assay. Table 1 shows that gentamicin or lysostaphin were able to inactivate all bacteria in the wells after 1 h incubation time. Subsequent lysis of HaCaT cells by addition of Triton did not yield any colonies in the plates, indicating that bacteria did not invade into the keratinocytes.

**Table 1:** Antibiotic invasion assay against *E. coli*, *S. aureus* ATCC 1803 and *S. aureus* ATCC 25923.

	CTL	CTL + HaCaT	S20	S20 + HaCaT	S30	S30 + HaCaT
***E. coli***						
PAS	6.16 ± 0.12	6.27 ± 0.09	ND	4.07 ± 0.43	ND	3.11 ± 0.08
Gentamicin	ND	ND	ND	ND	ND	ND
1% Triton	ND	ND	ND	ND	ND	ND
***S. aureus* ATCC 1803**						
PAS	5.93 ± 0.34	6.31 ± 0.13	ND	2.91 ± 0.19	ND	ND
Lysostaphin	ND	ND	ND	ND	ND	ND
1% Triton	ND	ND	ND	ND	ND	ND
***S. aureus* ATCC 25923**						
PAS	6.30 ± 0.05	6.46 ± 0.07	ND	3.68 ± 0.47	ND	2.83 ± 0.16
Lysostaphin	ND	ND	ND	ND	ND	ND
1% Triton	ND	ND	ND	ND	ND	ND

Results are the surviving bacterial concentration in log CFU/mL shown as mean ± S.D. of three determinations. ND = not detected, limit of detection 2 log.

### Comparison of PAS to disinfectants

The antimicrobial efficacy of seven different chemical disinfectants from different classes of disinfectants applicable to the biomedical field were compared with PAS. Higher concentrations of these disinfectants were also tested, but only the lowest concentrations are shown here. All solutions tested (1% povidone iodine, 1% Chlorhexidine, 2% formaldehyde, 2% glutaraldehyde, 0.045% NaClO, 70% ethanol and 3% H_2_O_2_) had strong antibacterial effects and were able to reduce *E. coli* or *S. aureus* to undetectable levels after 15 min contact time (Table 2). Strong cytotoxic effects were also observed for all disinfectants with cell viabilities below 20% for all solutions tested.

**Table 2:** List of the chemical disinfectants used and their antimicrobial activity and HaCaT cell viability after 15 min contact time.

Disinfectant used	Bacterial concentration in co-culture [CFU/mL]			Cell viability of HaCaT [%]
	*E. coli*	*S. aureus* ATCC 1803	*S. aureus* ATCC 25923	
CTL	6.26 ± 0.1	6.17 ± 0.1	6.46 ± 0.07	100 ± 0.1
PAS 20	6.12 ± 0.1	6.21 ± 0.1	5.87 ± 00.4	32.1 ± 6.8
1% Povidone Iodine	ND	ND	ND	14.7 ± 0.1
1% Chlorhexidine	ND	ND	ND	11.2 ± 0.3
2% Formaldehyde	ND	ND	ND	13.6 ± 0.3
2% Glutaraldehyde	ND	ND	ND	ND
0.045% NaClO	ND	ND	ND	9.6 ± 5.3
70% Ethanol	ND	ND	ND	17.5 ± 0.7
3% H_2_O_2_	ND	ND	ND	16.1 ± 1.0

ND = not detected, limit of detection 2 log.

### Decrease of cell viability and cell death of HaCaT cells induced by PAS

**Figure 5:**
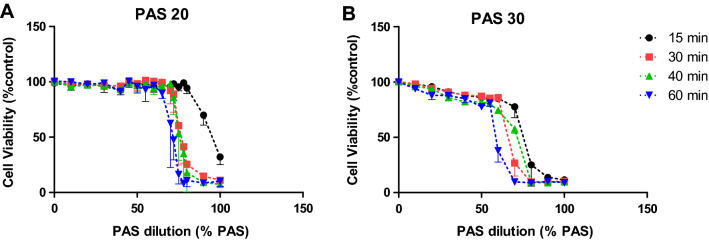
Cell viability of HaCaT cells treated with different dilutions of PAS 20 (A) and PAS 30 (B) at 15 min, 30 min, 40 min and 60 min contact time.

The resazurin assay was used to measure cell metabolic activity which served as an indicator of mammalian cell cytotoxicity. Pure plasma activated saline demonstrated strong cytotoxic effects on HaCaT cells, where the intensity of effects was a function of contact time duration and PAS concentration. Regarding PAS 20, 30% cells remained viable when incubated for 15 min with the undiluted PAS (100% PAS 20), whereas 30, 40 and 60 min contact time had stronger cytotoxic effects and no viable cells were observed. The effect of diluting PAS with PBS was therefore tested (Fig. 5A). Generally, cytotoxicity increased as a function of PAS concentration. Figure 5B shows that 75% cytotoxicity on HaCaT cells occurred following incubation of 80% PAS 30 for 15 min and a 90% reduction in viability when incubated for longer contact time. For 60 min contact time, 60% PAS 30 caused 60% cytotoxicity and 70% PAS 30 resulted in complete loss of cell viability. Comparing diluted PAS 20 and PAS 30, PAS 30 seems to follow the same trendline as PAS 20, but with a 20% PAS dilution difference. For instance, for 15 min contact time, cytotoxic effects start around 70% PAS 30 and 90% PAS 20 which both led to cell viability of 70%.

**Figure 6:**
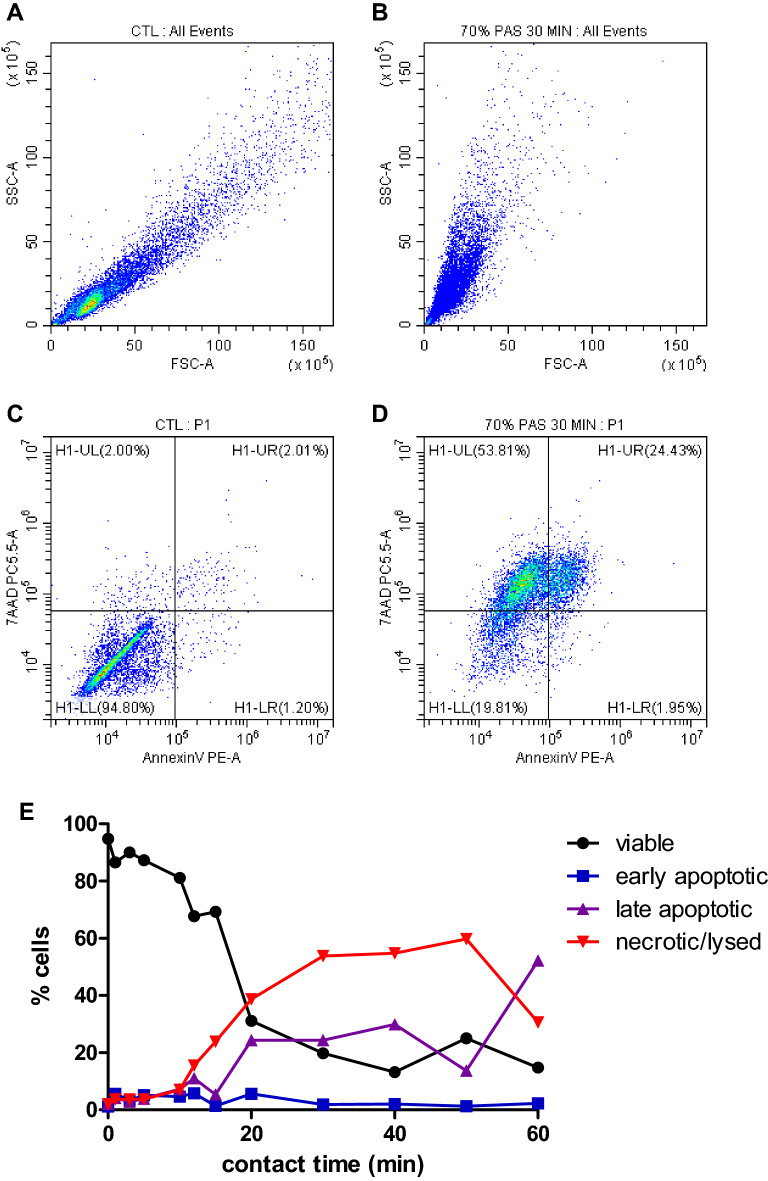
Apoptosis and lysis of HaCaT cells after incubation with 70% PAS at different contact times assessed using AnnexinV and 7AAD staining and flow cytometric analysis. Representative flow cytometry images showing FSC/SSC for control (**A**) and cells treated with 70% PAS for 30 min (**B**) and AnnexinV/7AAD staining for control (**C**) and 70% PAS 30 min (**D**). Representative progression of apoptosis/lysis over contact time is shown in E (viable: AnnV−/7AAD−, early apoptotic: AnnV+/7AAD−, late apoptotic: AnnV+/7AAD+, necrotic/lysed: AnnV-/7AAD+).

We further explored the cell death of HaCaT cells after incubation with different % of PAS from 1 min up to 60 min contact time by flow cytometric analysis (Fig. 6 and Table 1 Supplementary material). The flow cytometry data showed that at low concentrations of PAS, HaCaT cells maintained high levels of viability, however, when these cells were treated with higher concentrations of PAS accelerated cell lysis occurred, probably due to acidic pH (Table 1 Supplementary material). In more detail, when HaCaT were incubated with 55% PAS a maximum 2% of cells appeared as Annexin V-/7AAD+ after 60 min of incubation compared to control and a slight increase of AnnexinV+/7AAD− and AnnexinV+/7AAD+ (less than 5%) occurred. Generally, early apoptotic cell populations did not increase above 10% fraction for most of the samples, whereas the increase of late apoptotic and necrotic/lysed cells was higher. PAS concentrations such as 65% PAS led to > 20% late apoptotic cells and 40% lysed cells after 60 min contact time. 70% PAS was more cytotoxic and acidic and caused > 20% late apoptotic cells and 50% lysed cells within the shorter contact time of 30 min (Fig. 6 and Table 1) and even shorter contact times (15 min) caused similar effects using a 75% PAS solution. Changes in forward scatter/side scatter profile and microscopic observation indicated that the AnnexinV+/7AAD+ (normally considered late apoptotic) and AnnexinV−/7AAD+ populations represented severely damaged/lysed cells. The lack of progression of cells through the early apoptotic stage suggests that Annexin V+/7AAD+ cells are the result of phosphatidylserine on the inner side of the membrane becoming accessible for AnnexinV due to excessive membrane damage rather than an apoptotic pathway.

**Figure 7:**
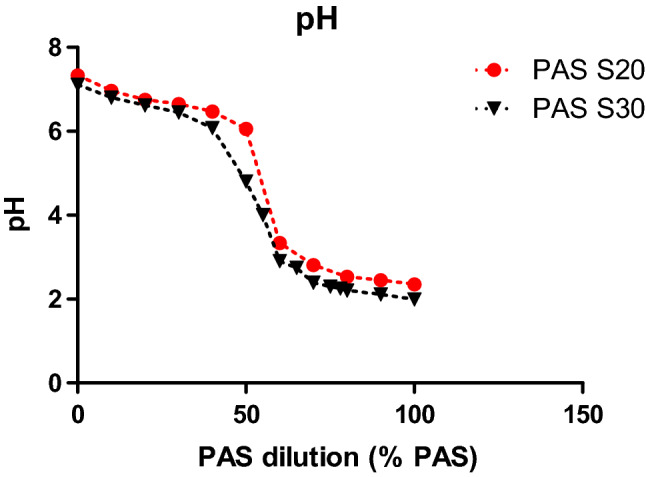
pH values of diluted PAS 20 and PAS 30.

**Figure 8:**
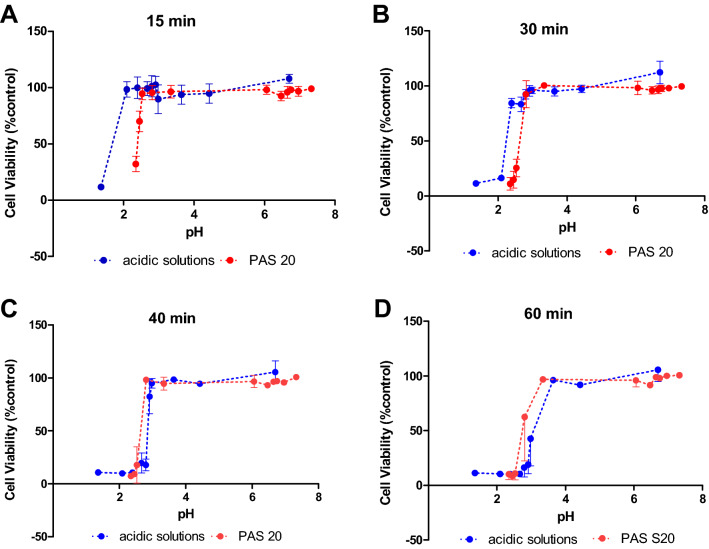
Cell viability of HaCaT cells in response to treatment with acidic solutions at pH values similar to pH values observed for PAS 20, compared with different dilutions of PAS 20.

**Figure 9:**
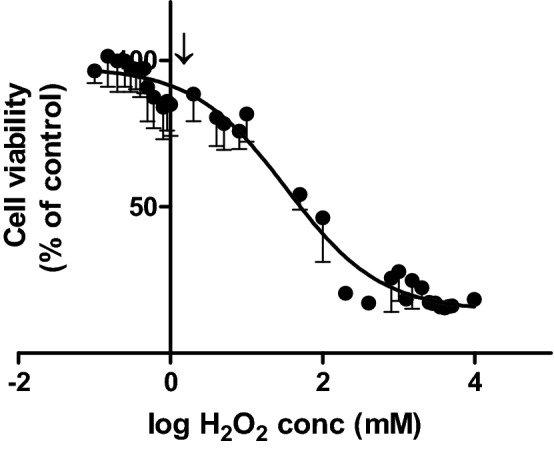
Cell viability of HaCaT cells at different concentrations of hydrogen peroxide after 60 min contact time, arrow indicates H_2_O_2_ concentration in PAS.

**Figure 10:**
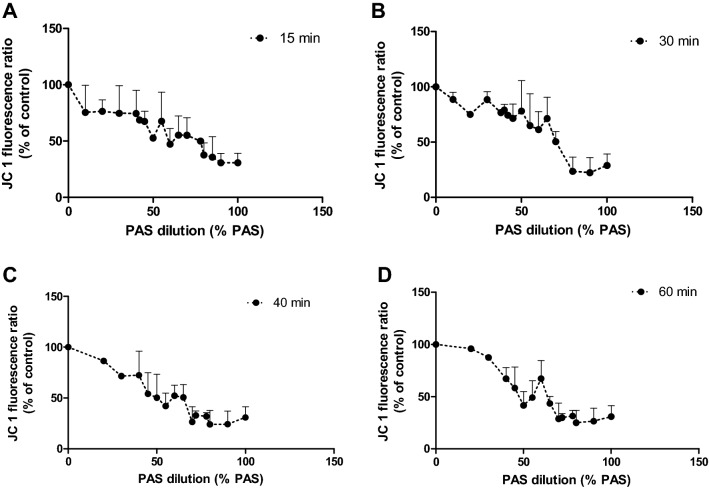
Mitochondrial membrane potential after incubation with PAS 20 for different contact times: (**A**) after 15 min, (**B**) 30 min, (**C**) 40 min, (**D**) 60 min contact time. Graphs represent averages of three independent PAS treatments.

**Figure 11:**
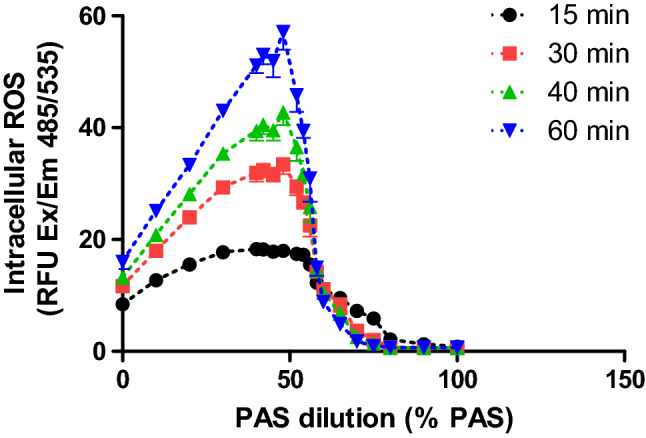
Detection of intracellular ROS after incubation of HaCaT with PAS 20 at different contact times. One representative experiment is represented.

**Figure 12:**
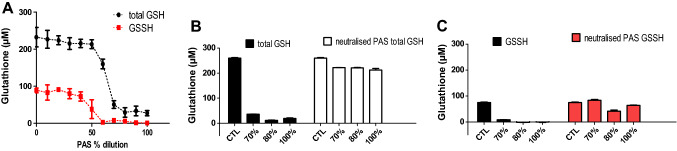
Measurements of total GSH and GSSH after 60 min exposure to PAS 20 and neutralized PAS.

### Extracellular pH as an important parameter influencing cell viability

The low pH value of PAS seems to play a vital role in the cytotoxic effects of less diluted samples as seen in the loss of cell viability at PAS concentrations above 60% in Figs. 5 and 6 which coincides with a sharp drop in the pH of the solution as it becomes more concentrated (Fig. 7). Generally, the more diluted samples had higher pH values, which were also a function of plasma generation time, with PAS 30 dilutions being slightly more acidic than PAS 20 dilutions (Fig. 7). However, pH level is only one aspect of the cytotoxicity mechanism, as shown with the pH controls. 100% PAS 20 with pH 2.35 was more cytotoxic than the acid control solution with pH of 2.39 for 15 and 30 min contact time (Fig. 8A, B). Extended contact times of 40 min and 60 min had similar effects for both PAS and acidic control solutions (Fig. 8C, D). Similar effects were observed for 90% PAS 20 and 80% PAS 20 with pH values 2.45 and 2.53, respectively, and both had higher cytotoxic effects than the pH control solution of pH 2.39. Acidic pH therefore potentiated the effects in cytotoxicity but the role of ROS and RNS cannot be excluded. Cell viability response to hydrogen peroxide was investigated to understand if hydrogen peroxide was responsible for the cytotoxic effects. The IC50 of hydrogen peroxide within the current setup with a contact time of 60 min was 33.57 mM (Fig. 9). A hydrogen peroxide concentration up to 10 mM, which is almost 5–6 times higher than PAS 20 or PAS 30, was found not to be cytotoxic, indicating that hydrogen peroxide on its own is not able to cause comparable cytotoxicity.

More diluted samples which were less cytotoxic than the undiluted samples, had less acidic pH levels, but were also diluted with regards to their chemical composition.

### Oxidative stress analysis

To further investigate the sharp loss in cell viability at PAS concentrations above 60%, analyses of cellular stress were performed including mitochondrial membrane potential, intracellular ROS levels and redox state in response to varying PAS concentrations. The JC-1 ratio of aggregates/monomers decreased as PAS 20 dilution decreased, with a peak happening around 55–60% PAS 20, indicating that at these dilutions, the cell membranes may be disrupted and aggregate components of mitochondria were exposed extracellularly (Fig. 10). An increase of intracellular ROS in cells, with a peak at 50% PAS for all contact times occurred and then a decrease followed as PAS concentration increased. Similar trendlines occurred for all contact times, with longer contact times showing higher fluorescence values (Fig. 11). The level of lipid oxidation following treatment with dilutions of PAS between 10% PAS and 100% PAS with 10% increments, over a range of contact times (10–60 min) was investigated. The levels of MDA did not increase (results not shown), and therefore lipid peroxidation was not detected for any contact time or solution. The same treatment was followed for analysis of glutathione and Fig. 12 shows that cells treated with up to 50% PAS have similar values for both total glutathione and oxidised glutathione (GSSH), with total glutathione (230 μM) having double concentration than GSSH (Fig. 12). Contact with 60% PAS showed a decrease of total glutathione to 160 μM with further decrease as concentration increased. Similarly, GSSH levels decreased to half after incubation with 50% PAS (37 μM), and lead to undetectable levels following more concentrated PAS. In order to investigate if reduction of GSH and GSSH was due to acidic pH, 70–100% PAS were neutralized with NaOH and results showed that total GSH and GSSH concentration were similar to controls, indicating that acidic pH could cause lysis to cells.

In summary these data demonstrate a role for oxidative stress at low PAS concentrations where the pH is neutral and support the observations from the previous sections that PAS concentrations above 60% result in cell lysis as a result of low pH.

## Discussion

For a good wound healing response, the bacterial load of wounds needs to be optimally managed, as infection can delay the process of wound healing. The primary objective of this study was to determine plasma treatment conditions which would provide a balance between cytotoxic and antibacterial effects of PAS in a co-culture set up, as a simplified model of infection control in a wound setting. In the current study, we confirm that PAS has strong antibacterial effects against both Gram negative and positive bacteria. Bacterial loads of all 3 strains used in this study were reduced depending on the plasma treatment time of the solution and contact time of liquids with the cells. Most importantly, PAS showed strong antibacterial effects (a reduction of at least 99.9% (≥ 3 log10)) against bacteria in the co-culture setup and especially *S. aureus*, where a 3–6 log reduction was achieved after 60 min depending on the strain.

Different bacterial species included in this study behaved differently to PAL. For example, *S. aureus* ATCC 1803 was inactivated by 6 log in the co-culture model after incubation for 60 min with PAS containing hydrogen peroxide and nitrates while only 2 log reduction was achieved for *E. coli*. Hozak et al. showed that PAW inhibits *S. epidermidis* more efficiently than *E. coli*^34^. Antibacterial effects of PAL are due to synergistic effects of acidic pH and reactive chemical species such as hydrogen peroxide, nitrite and nitrate^21^ and other unknown species which might be difficult to detect, and they can cause different effects on different types of bacteria.

The development of the co-culture, encompassing the growth of monolayers of HaCaT cells and addition of a planktonic bacterial population, enabled the investigation of the antibacterial potential of PAS in a more challenging environment. This co-culture method could be developed further into a more realistic infected wound model consisting of necrotic cells and biofilms of different bacteria since bacterial inactivation is more complex and difficult in biofilms^35^.

Studies have reported that antiseptics at specific concentrations such as povidone iodine solution may be beneficial in reduction of bacterial load in open wounds without trauma to the cells critical to wound repair^36^. On the other side, some antimicrobial agents have been shown to predispose to sensitization, such as neomycin which highly predisposes patients to contact allergy^37^ and alcohol-based hand sanitizers which can induce irritant contact dermatitis and skin burns^38^, while trace amounts of chlorhexidine gluconate can be absorbed through the skin^39^. PAS efficacy was compared with common chemical disinfectants and these were all more effective at shorter contact times, nonetheless they all had similar cytotoxic effects to PAS. Lee et al. reported comparison between cold plasma technology and peracetic acid and showed that peracetic acid had much higher and faster reduction rates^40^. Interestingly, in vivo experiments in mice showed that PAW treatment can be more efficient on wound healing processes than medical alcohol, by the promotion of the release of pro-inflammatory factors and anti-inflammatory factors in the wound^41^. Combining these data together, it seems that in the near future, PAL may still become a novel antimicrobial solution in the field of hygiene.

While hydrogen peroxide was detected in PAS, the cytotoxic effects of hydrogen peroxide solution were not similar to PAS and supplementation of cell cultures with nitrite or nitrate did not show cytotoxic effects in studies performed by others^42,43^. These observations suggest that another factor such as acidic pH plays a crucial role in the effects observed here, with cell viability being decreased by more than 50% after incubation with PAS with pH 2.53–2.81. HaCaT cells were incubated with PAS for a maximum time of one hour, a time which was not enough for H_2_O_2_ to cause cytotoxic effects.

A general indication of the redox state of the cells was given by studying the changes in intracellular ROS, mitochondria depolarisation, GSH and lipid peroxidation after exposure of HaCaT to PAS. Results revealed that intracellular ROS was enhanced, and breakdown of mitochondrial membrane potential followed which was associated with slight increases in apoptosis for PAS concentrations below 50%. These results indicate that low % of PAS are responsible for oxidative stress through increased intracellular ROS levels and the breakdown of mitochondrial membrane potential. A peak of fluorescence for intracellular ROS was observed around 50% PAS with a sharp drop and complete loss of ROS signal for higher concentrations of PAS. Under the same conditions cell viability values were low too, suggesting that dead/lysed cells cannot produce intracellular ROS. Similar results have been obtained in studies investigating direct plasma exposure of fibroblasts and keratocytes^44^. Also, PBS treated for 5 min with either He plasma or He-N_2_ plasma and predominant species H_2_O_2_ and nitrites lead to death in HaCaT cells after 1 h of exposure^45^.

Cells maintain a reducing intracellular environment to avoid genomic damage after induction of oxidative stress. Glutathione is the most important redox regulatory factor^46^ and its oxidation to glutathione disulfide (GSSG) is associated with oxidative stress. GSH depletion produced by different ways generates a low GSH/GSSG ratio leading to oxidative stress and apoptosis of cells by exacerbation of reactive oxygen species production^47^. HaCaT cells are able to express the main enzymes involved in the glutathione cycle in a way that is comparable to that observed in healthy human skin^48^. Following application of a non-toxic concentration of PAS (lower or equal to 60%), the amount of total GSH was not depleted and levels were maintained similar to control, demonstrating an active GSH cycle in HaCaT cells. Incubation with PAS equal or higher than 70% caused an immediate decrease in total cellular glutathione concentration GSH with levels significantly lower than control. According to Klinkhammer et al., DBD plasma treatment on GSH resulted in oxidation of the sulphur atoms from thiols to S–O, S = O, S–S and S–N = O groups, resulting in GSO_3_H, GSSG and GSNO. GSO_3_H was one of the most dominant final products which accumulated with longer plasma treatment times, whereas GSSG was the most prevailing molecule only after short treatment^49^. According to this theory, it is possible that decrease of GSH and GSSH at 60–70% PAS is due to transformation of these molecules to others such as GSO_3_H after incubation with more concentrated solutions. The GSH decrease could be largely prevented by neutralization of the acidic pH through addition of NaOH, indicating that the dominant reactions are influenced by pH value^49^. Consideration needs to be taken, that treatments were carried out for a maximum of 60 min, thus possible rupture of plasma membranes, and cell lysis with leakage of cell components such as glutathione might have happened and glutathione may have been lost during washing steps.

Lipid peroxidation caused by reactive species can lead to pore formation in the membrane and facilitate the diffusion of RONS into the cell. No increase in lipid peroxidation was detected by analyzing the accumulation of MDA, suggesting that changes in cell morphology were not due to oxidation of the membrane lipid. Lipid peroxidation in HaCaT cells was also not increased after exposure to three plasma devices in other studies, even if increase in DNA damage was detected^45^. In case of cancer cell lines such as melanoma and glioblastoma cells, PAM or direct exposure to plasma, respectively, caused an increase in the formation of MDA in cells^50,51^.

## Conclusion

Taken together, our results suggest that PAS is highly antimicrobial in the co-culture model but leads to changes in HaCaT cells with increase of intracellular ROS as early sign of apoptosis, then breakdown of the mitochondrial membrane potential and subsequent cell lysis and leakage of intracellular components such as GSH as concentration of PAS increases. We conclude that if PAS is diluted enough (i.e., 40–50%), cell viability remains unaffected with a minimal increase in early apoptotic cells, whereas undiluted PAS negatively influences the number of healthy cells with undesired effects of cell lysis. Our results demonstrate the difficulty in establishing a balance between antimicrobial efficacy and low cytotoxic effects since wound related cells such as HaCaT are affected negatively by incubation with PAS earlier than inactivation of bacteria. These findings may have implications for refining plasma activated liquids towards the design of a highly antimicrobial solution with low or moderate cytotoxic effects in surrounding tissues. While the single application of PAS may be of limited efficacy, improved delivery methods or repeated shorter applications may enhance the antimicrobial effects while reducing detrimental effects on eukaryotic cells. Additional studies are required for determining the mechanism of action involved in the wound healing activity, and the elucidation of other chemical species in PAS in order to validate if PAS could be used in the field of hygiene as a novel antimicrobial agent.

## Supplementary Information

Supplementary Table 1.
