# Supplementary material for: Efficacy of plasma activated saline in a co-culture infection control model
Source: Sci Rep. 2022 Nov 23;12:20230. doi: 10.1038/s41598-022-20165-z (PMC9684424; doi:10.1038/s41598-022-20165-z)
Supplement: Supplementary file 1 — Supplementary Table 1. [file 41598_2022_20165_MOESM1_ESM.docx]

Supplementary Table 1. Cell death analysis of HaCaT cells incubated with different concentrations of PAS (55%-75%) for 1-60 min based on staining with AnnexinV and PI and flow cytometric analysis.

| **Contact time (min)** | | | | | | | | | | | | | |
| --- | --- | --- | --- | --- | --- | --- | --- | --- | --- | --- | --- | --- | --- |
|  | **0** | **1** | **3** | **5** | **10** | **12** | **15** | **20** | **30** | **40** | **50** | **60** |  |
| **Cell percentages (%)** | | | | | | | | | | | | | |
| **PAS 55%** | | | | | | | | | | | | | |
| AnnV-/7ADD-  Viable cells | 86.7 | 78.8 | 87.9 | 93.9 | 92.6 | 91.3 | 92.7 | 89.4 | 95.1 | 91.3 | 86.5 | 91.3 |  |
| AnnV+/7ADD-  Early apoptotic | 10.3 | 6.4 | 7.3 | 3.4 | 3.3 | 3.2 | 3.8 | 5.8 | 2.5 | 5.4 | 6.5 | 3.7 |  |
| AnnV+/7ADD+  Late apoptotic | 2.1 | 12.8 | 3.6 | 1.4 | 2.9 | 3.2 | 2.5 | 3.2 | 1.2 | 2.2 | 5.0 | 3.1 |  |
| AnnV-/7ADD+  Necrotic/Lysed | 0.8 | 1.9 | 1.0 | 1.3 | 1.1 | 2.1 | 0.8 | 1.5 | 1.1 | 1.0 | 1.9 | 1.8 |  |
| **PAS 60%** | | | | | | | | | | | | | |
| AnnV-/7ADD-  Viable cells | 81.7 | 87.4 | 83.0 | 84.7 | 81.6 | 87.7 | 74.7 | 83.8 | 92.0 | 87.0 | 92.7 | 88.0 |  |
| AnnV+/7ADD-  Early apoptotic | 14.0 | 7.4 | 11.4 | 8.1 | 12.6 | 5.9 | 2.7 | 5.5 | 4.1 | 7.6 | 4.3 | 7.1 |  |
| AnnV+/7ADD+  Late apoptotic | 2.5 | 3.1 | 3.5 | 4.3 | 3.5 | 4.1 | 6.6 | 7.4 | 2.3 | 3.6 | 1.6 | 2.3 |  |
| AnnV-/7ADD+  Necrotic/Lysed | 1.8 | 1.8 | 2.0 | 2.7 | 2.2 | 2.1 | 16.0 | 3.2 | 1.5 | 1.8 | 1.3 | 2.5 |  |
| **PAS 65%** | | | | | | | | | | | | | |
| AnnV-/7ADD-  Viable cells | 84.5 | 66.9 | 81.9 | 88.1 | 88.2 | 87.9 | 85.2 | 80.3 | 61.2 | 58.4 | 56.2 | 33.1 |  |
| AnnV+/7ADD-  Early apoptotic | 2.4 | 12.2 | 7.6 | 3.9 | 1.5 | 2.8 | 4.7 | 4.5 | 3.8 | 5.0 | 7.6 | 3.4 |  |
| AnnV+/7ADD+  Late apoptotic | 4.4 | 13.2 | 6.0 | 4.1 | 3.5 | 3.8 | 6.5 | 9.7 | 9.6 | 17.3 | 18.4 | 23.6 |  |
| AnnV-/7ADD+  Necrotic/Lysed | 8.5 | 7.5 | 4.4 | 3.8 | 6.7 | 5.4 | 3.1 | 5.3 | 25.2 | 19.3 | 17.8 | 39.7 |  |
| **PAS 70%** | | | | | | | | | | | | | |
| AnnV-/7ADD-  Viable cells | 94.8 | 86.5 | 90.0 | 87.3 | 81.1 | 67.7 | 69.3 | 31.1 | 19.8 | 13.2 | 25.0 | 14.8 |  |
| AnnV+/7ADD-  Early apoptotic | 1.2 | 5.6 | 3.3 | 5.1 | 4.6 | 5.8 | 1.4 | 5.6 | 1.9 | 2.0 | 1.3 | 2.3 |  |
| AnnV+/7ADD+  Late apoptotic | 2.0 | 4.1 | 2.9 | 3.9 | 7.3 | 11.0 | 5.4 | 24.4 | 24.4 | 29.9 | 13.7 | 52.2 |  |
| AnnV-/7ADD+  Necrotic/Lysed | 2.0 | 3.7 | 3.7 | 3.7 | 7.0 | 15.5 | 23.9 | 38.7 | 53.8 | 54.8 | 59.8 | 30.6 |  |
| **PAS 75%** | | | | | | | | | | | | | |
| AnnV-/7ADD-  Viable cells | 82.2 | 71.1 | 77.6 | 40.1 | 49.2 | 20.4 | 22.6 | 28.9 | 26.6 | 32.8 | 11.4 | 15.1 |  |
| AnnV+/7ADD-  Early apoptotic | 7.2 | 6.0 | 8.6 | 15.3 | 1.6 | 2.0 | 2.4 | 5.2 | 3.9 | 2.4 | 0.1 | 0.7 |  |
| AnnV+/7ADD+  Late apoptotic | 5.5 | 9.6 | 6.9 | 34.2 | 10.8 | 12.7 | 19.6 | 20.3 | 17.7 | 11.6 | 19.0 | 15.1 |  |
| AnnV-/7ADD+  Necrotic/Lysed | 5.0 | 13.2 | 6.9 | 10.3 | 38.2 | 64.8 | 55.3 | 45.6 | 51.8 | 53.1 | 68.7 | 69.1 |  |
